# Novel Calcium-Binding Motif Stabilizes and Increases the Activity of *Aspergillus fumigatus* Ecto-NADase

**DOI:** 10.1021/acs.biochem.3c00360

**Published:** 2023-11-07

**Authors:** Eugenio Ferrario, Juha P. Kallio, Øyvind Strømland, Mathias Ziegler

**Affiliations:** † Department of Biomedicine, University of Bergen, Jonas Lies vei 91, Bergen 5009, Norway; ‡ Leibniz Institute for Natural Product Research and Infection Biology, Hans Knöll Institute, Beutenbargstraße 11A, Jena 07745, Germany

## Abstract

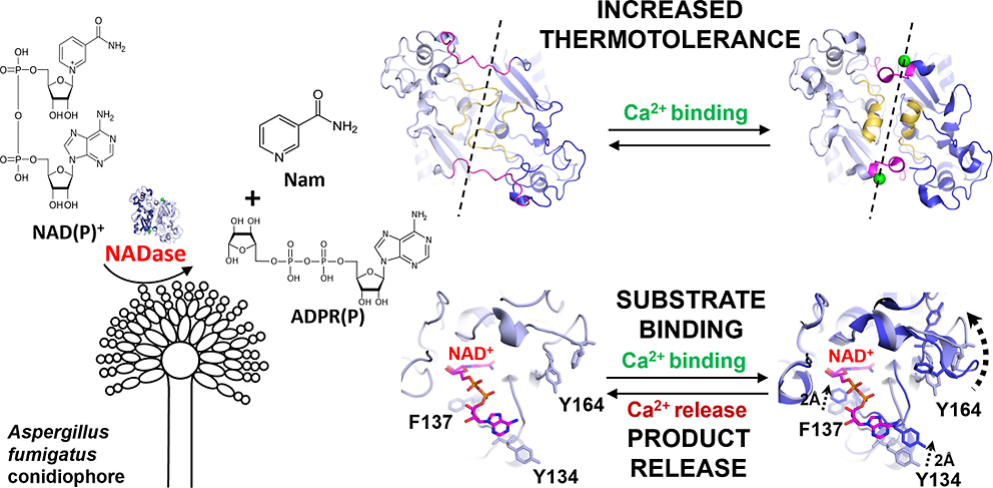

Nicotinamide adenine dinucleotide (NAD) is an essential molecule in all kingdoms of life, mediating energy metabolism and cellular signaling. Recently, a new class of highly active fungal surface NADases was discovered. The enzyme from the opportunistic human pathogen *Aspergillus fumigatus* was thoroughly characterized. It harbors a catalytic domain that resembles that of the tuberculosis necrotizing toxin from *Mycobacterium tuberculosis*, which efficiently cleaves NAD^+^ to nicotinamide and ADP-ribose, thereby depleting the dinucleotide pool. Of note, the *A. fumigatus* NADase has an additional Ca^2+^-binding motif at the C-terminus of the protein. Despite the presence of NADases in several fungal divisions, the Ca^2+^-binding motif is uniquely found in the Eurotiales order, which contains species that have immense health and economic impacts on humans. To identify the potential roles of the metal ion-binding site in catalysis or protein stability, we generated and characterized *A. fumigatus* NADase variants lacking the ability to bind calcium. X-ray crystallographic analyses revealed that the mutation causes a drastic and dynamic structural rearrangement of the homodimer, resulting in decreased thermal stability. Even though the calcium-binding site is at a long distance from the catalytic center, the structural reorganization upon the loss of calcium binding allosterically alters the active site, thereby negatively affecting NAD-glycohydrolase activity. Together, these findings reveal that this unique calcium-binding site affects the protein fold, stabilizing the dimeric structure, but also mediates long-range effects resulting in an increased catalytic rate.

## Introduction

Nicotinamide adenine dinucleotide (NAD) is one of the essential molecules on which life is based. In humans, it mediates many bioenergetic and signaling pathways crucial for cell metabolism and cell cycle progression.^1^ NAD plays a vital role in redox reactions, where it is reduced to NADH and oxidized to NAD^+^. In signaling pathways, NAD^+^ is cleaved into nicotinamide (Nam) and an ADP-ribose (ADPR) derivative. In ADPR transferase reactions, most notably catalyzed by the PARP protein family, ADPR is transferred onto a substrate molecule, most commonly proteins.^2^ In NAD-dependent protein deacylation reactions catalyzed by Sirtuins, ADPR functions as an acceptor of an acyl group giving rise to O-acyl-ADPR.^3^ Given that NAD^+^ is consumed in these reactions, a constant resynthesis of the dinucleotide is needed. The majority of cellular NAD^+^ is generated in the salvage pathway, where the Nam released in signaling reactions is resynthesized into NAD^+^.^4^ Given its importance, NAD^+^ is targeted by toxins from many pathogenic microorganisms, including *Streptococcus pyogenes* and *Mycobacterium tuberculosis*. These toxins invade cells and act as NAD glycohydrolases, cleaving NAD^+^ into Nam and ADPR and rapidly depleting the cellular NAD pool, ultimately leading to cell death.^5−7^ In this context, we recently discovered a new fungal NADase on the surface of conidia from *Aspergillus fumigatus* (*Af*NADase),^8^ the predominant etiologic agent of Aspergillosis.^9^ Conidial NADase activity was first discovered in the 1950s in fungi of the Ascomycota division, most notably *Neurospora crassa*.^10^ Nevertheless, the gene and protein responsible for this activity were only recently identified.^8^ Earlier research focused on the role of the *N. crassa* NADase during aerial growth and conidiation.^11,12^ However, the physiological function of fungal surface NADases has remained elusive.

Even though they have not been conclusively demonstrated, fungal NADases may be potential virulence factors that can facilitate fungal infections. An indication supporting this hypothesis is the tuberculosis necrotizing toxin (TNT) from *Mycobacterium tuberculosis* (Mtb). It is an enzyme that rapidly hydrolyzes NAD^+^ in Mtb-infected macrophages,^13^ causing a necrotic-like cell death helping Mycobacterium evade the immune response of the infected host. The catalytic part of the TNT is structurally highly similar to *Af*NADase. The crystal structure of TNT (PDB ID: 4QLP) has been solved in complex with its immunity factor (IFT)^14^ that inhibits the enzyme while inside *Mycobacterium*. The *Af*NADase has been purified as an active homodimer which was also confirmed by determining the crystal structure (PDB ID: 6YGE).^8^ Intriguingly, the active site architecture of the bacterial and fungal enzymes is conserved, but their structural superimposition reveals the presence of a unique C-terminal calcium-binding motif in *Af*NADase, which is located at the edge of the dimerization interface. Therefore, it may represent a novel means to stabilize a dimer. On the other hand, omitting or chelating calcium during enzymatic measurements lowered NADase activity despite the relatively long distance (∼25 Å) between the calcium ion and the catalytic site.^8^ Consequently, calcium binding may affect catalysis through indirect mechanisms.

In the present study, we addressed the structural and functional role of calcium binding by introducing mutations in the C-terminus of *Af*NADase that precluded the binding of the metal ion. Our analyses indicate that the Ca^2+^-binding motif of the enzyme has a major impact on both the thermostability of the protein and the catalytic activity. Based on the observed alterations in the three-dimensional structure, we propose a novel mechanism of enzyme regulation based on long-range interactions mediated by calcium binding.

## Results

### *Af*NADase Ca^2+^ Binding Motif Affects Catalytic Activity and Protein Stability

**Figure 1:**
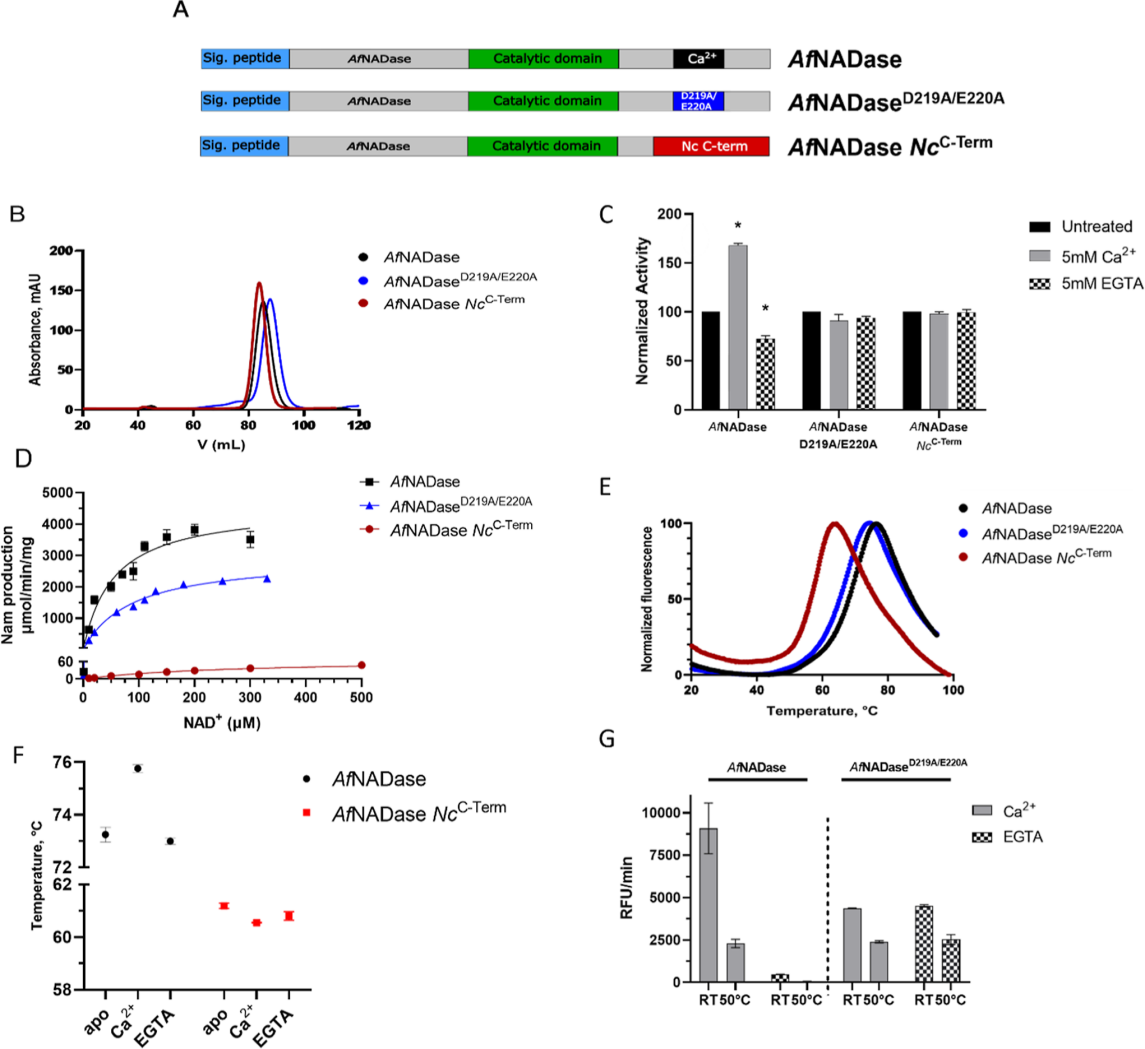
Biochemical and biophysical alterations of NADase Ca^2+^motif variants. (A) Domain architecture of *Af*NADase, *Af*NADase^D219A/E220A^, and *Af*NADase *Nc*^C-Term^. (B) Size exclusion chromatography profile of purified NADases. (C) EGTA/calcium sensitivity of purified *Af*NADase variants. *n* = 2 assay was performed in triplicate. Data is normalized using the activity of each untreated enzyme as a reference. Error bars represent the standard deviation. *p-value <0.05 (D) kinetic analysis of *Af*NADase (*K*_M_ = 54.8 ± 15.3 μM *V*_max_ = 4574 ± 458 μmol/min/mg), *Af*NADase^D219A/E220A^ (*K*_M_ = 88.3 ± 12.2 μM *V*_max_ = 2956 ± 157 μmol/min/mg), and *Af*NADase *Nc*^C-Term^ (*K*_M_ = 272.8 ± 48.6 μM *V*_max_ = 69.8 ± 5.3 μmol/min/mg) using a fluorometric assay and etheno-NAD^+^ as substrate. *n* = 2 assay was performed in triplicate. Results are summarized in Table 1 together with *T*_M_ values. Error bars represent the standard deviation. (E) DSF profiles of the different *Af*NADase variants with corresponding melting temperature values reported in the table together with the kinetic parameters of each enzyme. *n* = 3 assay was performed in triplicate; the graph shows a representative experiment. (F) DSF-derived *T*_M_ values of *Af*NADase and *Af*NADase *Nc*^C-Term^ in the presence of Ca^2+^ or EGTA. (*Af*NADase = apo: 73.25 °C ± 0.27, Ca^2+^:75.75 °C ± 0.14, EGTA: 72.99 °C ± 0.11; *Af*NADase *Nc*^C-Term^ = apo: 61.19 °C ± 0.09, Ca^2+^:60.55 °C ± 0.01, EGTA: 60.80 °C ± 0.16); *n* = 2 assay was performed in triplicate. Error bars represent the standard deviation. (G) Residual NADase activity after incubation at 50 °C for 10 min with Ca^2+^ or EGTA. *n* = 2 assay was performed in triplicate. Error bars represent the standard deviation.

To investigate the function of the Ca^2+^-binding motif in *Af*NADase, we generated two variants. In the first variant, we abolished calcium binding by mutating two critical residues for metal ion coordination into alanine (D219A/E220A), referred to here as *Af*NADase^D219A/E220A^. In the second variant, we created a chimeric NADase by replacing the 10 C-terminal amino acids, including the Ca^2+^-binding motif, with the C-terminus from *N. crassa* NADase (*Nc*NADase). Importantly, *Nc*NADase lacks the calcium binding site and as a result the chimeric protein, here referred to as *Af*NADase *Nc*^C-Term^, is predicted to be unable to bind calcium (Figures 1A and S1A). The three enzyme variants, including *Af*NADase, were expressed in baculovirus-infected Sf9 insect cells and purified by affinity and size exclusion chromatography. Size exclusion chromatography did not show any drastic change in the elution profile of the three proteins, indicating that the mutations do not affect the oligomeric state of the enzyme (Figure 1B).

As expected, both variants lost sensitivity toward Ca^2+^ and EGTA, whereas *Af*NADase activity changed in the presence or absence of calcium (Figure 1C). These observations indicate that both variants indeed lack the ability to bind calcium. The kinetic profiles of the mutant enzymes differed substantially when compared to *Af*NADase when using the fluorescent NAD analogue etheno-NAD^+^ as substrate (Figure 1D and Table 1). For the wild-type *Af*NADase, we obtained a *K*_M_ of 54.8 (±15.3) μM and a *V*_max_ of 4574 (±458) μmol/min/mg. Compared to the wild-type enzyme, *Af*NADase *Nc*^C-Term^ showed a 5-fold decrease in affinity and a 66-fold reduced conversion rate (*K*_M_ of 272.8 ± 48.6 μM and a *V*_max_ of 69.8 ± 5.3 μmol/min/mg). Interestingly, the *K*_M_ (88.3 ± 12.2 μM) and *V*_max_ (2956 ± 157 μmol/min/mg) of *Af*NADase^D219A/E220A^ were far less affected than the *Af*NADase *Nc*^C-Term^. These results demonstrate that the inability to bind calcium affects the active site of NADase, linking the catalytic properties directly to calcium binding.

**Table 1:** Kinetic Parameters and *T*_M_ of *Af*NADase, *Af*NADase^D219A/E220A^, and *Af*NADase *Nc*^C-Term^

	*T*_M_ (°C)	*K*_M_ (μM)	*V*_max_ (μmol/min/mg)
*Af*NADase	73.25 ± 0.27	54.8 ± 15.3	4574 ± 458
*Af*NADase^D219A/E220A^	67.88 ± 0.16	88.3 ± 12.2	2956 ± 157
*Af*NADase *Nc*^C-Term^	61.19 ± 0.09	272.8 ± 48.6	69.8 ± 5.3

We considered the possibility that the differences in the kinetic parameters could be a consequence of a major structural reorganization of the enzyme. Therefore, we analyzed the thermostability of the constructs. Using differential scanning fluorimetry (DSF) we determined the melting temperature (*T*_M_) of the three NADase variants. Notably, the *T*_M_ values of *Af*NADase and *Af*NADase^D219AE220A^ differ only slightly. In contrast, *Af*NADase *Nc*^C-Term^ showed a large shift in *T*_M_, from 71.4 to 57.9 °C for the wild-type and *Nc*^C-Term^, respectively (Figure 1E and Table 1). These data suggest that at least in the *Af*NADase *Nc*^C-Term^, the overall stability of the protein is affected by the modification of the C-terminus that eliminates the calcium binding site. Following what is indicated in Figure 1C, we investigate the influence of Ca^2+^ on the thermal stability of the NADase. As shown in Figure 1F, thermal stability of *Af*NADase increases in the presence of Ca^2+^ (*T*_M_: 75.7 5 ± 0.14, + 2.5 °C), while no effect was noted for *Af*NADase *Nc*^C-Term^.

To further validate a possible impact of Ca^2+^-binding on the thermal stability of *Af*NADase, we measured the residual enzyme activity upon heat treatment in the presence or absence of Ca^2+^ ions. As shown in Figure 1G, the residual activity of *Af*NADase^D219AE220A^ following heat treatment is similar irrespective of the presence of Ca^2+^. In contrast, *Af*NADase was significantly more active when the heat treatment was conducted in the presence of Ca^2+^ (Ca^2+^ RT/50 °C = 3.5; EGTA RT/50 °C = 7.5), suggesting that calcium binding contributes to protein stability.

### *Af*NADase^D219A/E220A^ Structure Reveals a Dynamic Rearrangement of the Protein Fold in the Absence of Calcium

**Figure 2:**
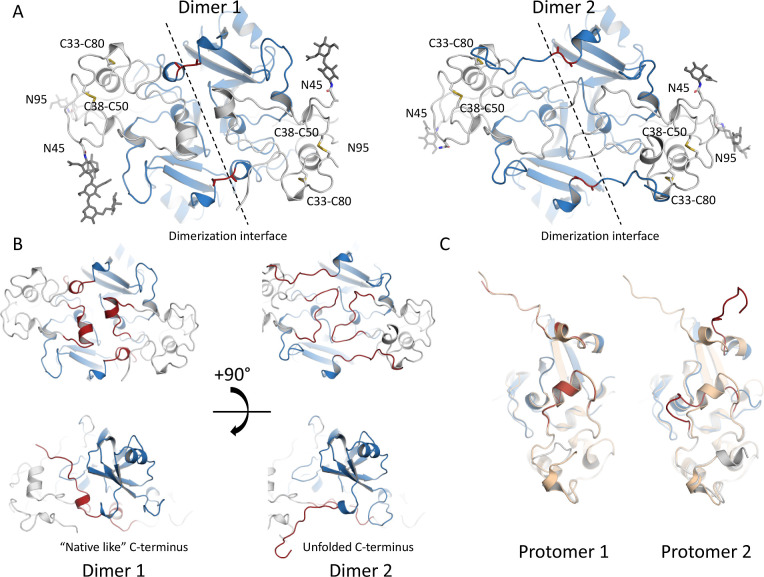
Cartoon representation of the two dimers present in the ASU of the crystal structure of *Af*NADase^D219A/E220A^. (A) Overall structure of dimers 1 and 2 (PDB ID: 8PMR). The catalytic domain is shown in blue and the palm domain in gray. Mutated amino acids Ala219 and Ala220 are shown in red. (B) Dimers 1 and 2 are shown with the helix in the dimerization interface and the C-terminus terminus is highlighted. The left panel shows a native-like conformation, and the right panel shows the formation of a loop-to-loop interaction in the dimerization interface as well as the unfolding of the C-terminus. (C) Comparison of a protomer from dimer 1 (left) and dimer 2 (right) to a protomer from a wild-type structure (PDB ID: 6YGE) is shown in light brown.

As demonstrated above, eliminating only the ability to bind Ca^2+^ in the *Af*NADase^D219A/E220A^ variant was sufficient to provoke considerable changes in the thermal stability as well as a decrease in the substrate affinity and catalytic rate. Therefore, we decided to investigate the structural basis for these changes at the atomic level, as this would allow us to elucidate the mechanism of how the calcium binding motif regulates NADase activity and stability. The protein was crystallized in space group *P*6_5_, and the crystal structure was solved to 1.94 Å resolution (Table 2) with four protomers in the asymmetric unit (ASU) (Figures 2 and S2, PDB ID: 8PMR). The ASU contained two homodimers, which were lacking Ca^2+^ ions as expected. Both dimers were assembled in a similar manner to the *Af*NADase (PDB ID: 6YGE). A closer inspection revealed that one dimer represented the native-like conformation, whereas the other had a conformation with an unfolded C-terminus (Figure 2A). To eliminate the possibility that differences between detected dimers would be due to crystallization, the analysis of the crystal packing was carried out. The crystal packing does not cause any clashes between the ASU and the symmetry-related molecules, and crystal contacts do not appear to induce steric forces that would drive the conformation to either a folded or unfolded C-terminus (Figure S2). The overall fold of the two homodimers resembles that of the wild-type structure. Superimposition of the dimers to *Af*NADase (C alpha) showed RMSD of 0.25 and 1.00 for chain AB (native-like) and chain CD (unfolded C-terminus), respectively. From here on the chain, the AB dimer is referred to as dimer 1 and the CD dimer as dimer 2.

**Table 2:** Data Collection and Refinement Statistics^a^

	*Af*NADase^D219/AE220A^	*Af*NADase *Nc*^C-Term^
Data Collection		
wavelength	0.976	1.033
resolution range	39.32–1.943 (2.012–1.943)	46.67–2.4 (2.486–2.4)
space group	*P*6_5_	*P*4_1_2_1_2
unit cell	65.897 65.897 488.199 90 90 120	101.018 101.018 366.335 90 90 90
multiplicity	11.6 (5.8)	8.7 (9.3)
completeness (%)	96.35 (67.14)	99.81 (99.91)
mean I/sigma(I)	18.85 (0.60)	14.38 (0.87)
wilson B-factor	39.97	73.61
*R*_merge_	0.0759 (2.11)	0.0816 (2.137)
*R*_pim_	0.0229 (0.885)	0.0296 (0.7324)
CC1/2	0.999 (0.275)	0.999 (0.456)
Refinement		
no of reflections/no of reflections for *R*_free_	84441 (4212)	75238 (3632)
*R*_work_/*R*_free_	0.182/0.216	0.202/0.223
number atoms		
protein	6597	6073
ligand	474	320
solvent	620	135
RMS(bonds)	0.010	0.009
RMS(angles)	1.11	1.41
Ramachandran favored (%)	98.19	97.46
Ramachandran allowed (%)	1.45	2.41
Ramachandran outliers (%)	0.36	0.13
average B-factor		
protein	47.77	85.16
ligand	75.39	119.85
solvent	49.64	74.95

a Statistics for the highest-resolution shell are shown in parentheses.

The *Af*NADase^D219A/E220A^ structure contains two disulfide bonds per protomer, located in the “thumb domain”, and the same N-linked glycosylation sites that were already observed in the wild-type structure (PDB ID:6YGE).^8^ The major differences between the two *Af*NADase^D219/AE220A^ dimers are located at the C-terminus and at the dimerization interface (Figure 2B). Dimer 1 has a C-terminus folded in a manner identical to that of *Af*NADase with the characteristic helix-turn-helix motif for the Ca^2+^-binding site, allowing the C-terminus to intertwine on the surface of the complementary protomer participating in dimer formation. In contrast, in dimer 2, the C-terminal region, including the mutated Ca^2+^-binding site, is unfolded, causing the C-terminus to turn ∼45° and lose the interactions with the complementary protomer of the dimer. Strikingly, the D219A/E220A mutations also caused a major rearrangement of the dimerization interface. The two α-helixes, forming part of the dimerization interface of *Af*NADase^8^ and dimer 1 of *Af*NADase^D219A/E220A^, are unfolded in dimer 2 and rather form a loop-to-loop interaction (Figure 2B,C). The comparison of both observed dimers to *Af*NADase shows that dimer 1 represents a fold essentially identical to the native conformation (Ca^2+^-bound state in *Af*NADase), whereas the conformation of dimer 2 is notably different, representing a dynamic counterpart with unfolded C-terminus and a major reorganization of the dimer interface. Our hypothesis is that this would represent the state of *Af*NADase in the absence of bound Ca^2+^.

### Changes in the *Af*NADase Structure upon Ca^2+^-Mediated C-Terminal Folding

**Figure 3:**
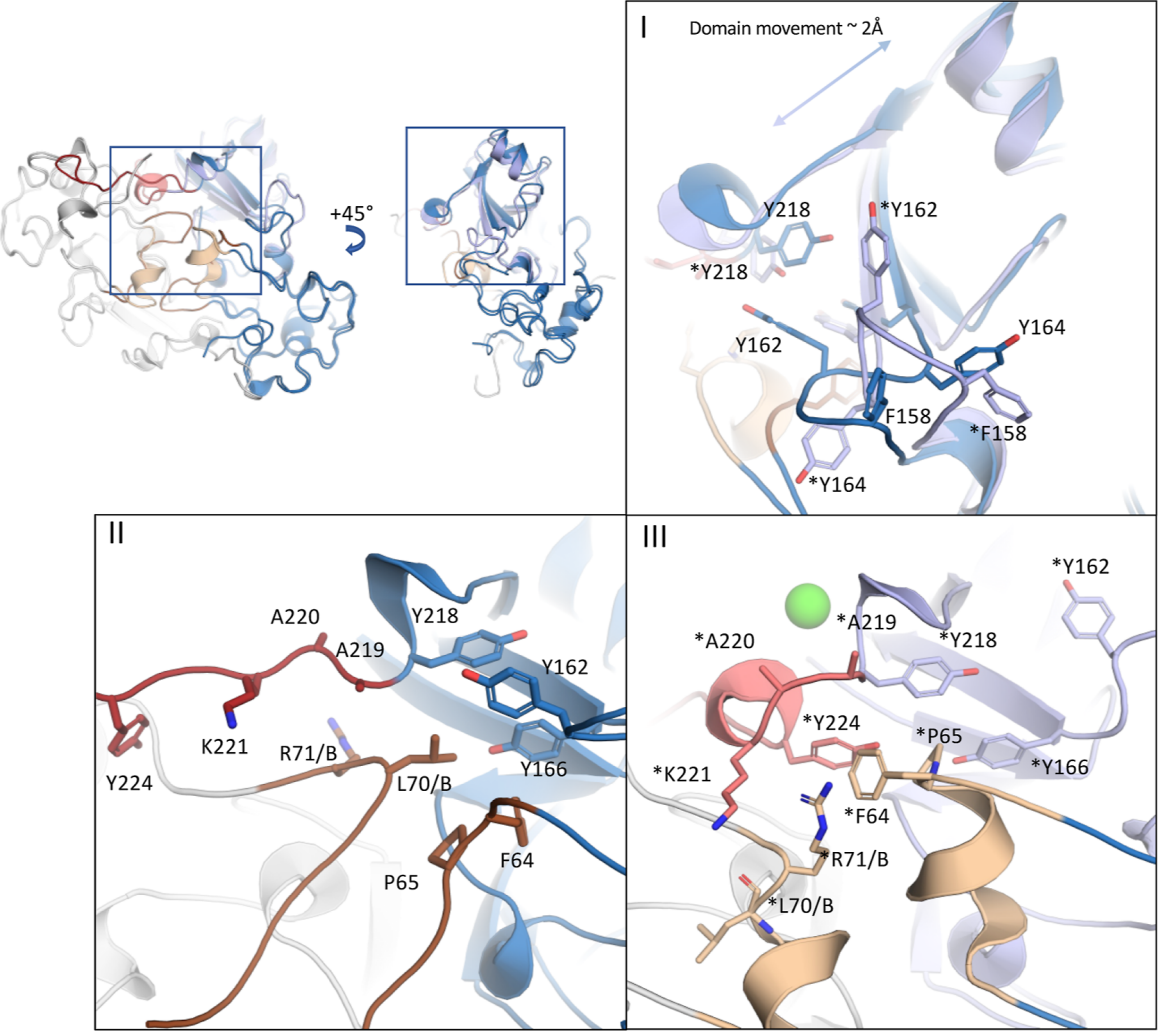
Details of the structural differences on the molecular level between dimers 1 and 2 of *Af*NADAase^D219A/E220A^. Superimposition of dimer 1 and dimer 2 (PDB ID: 8PMR) structures showing conformational changes at the dimerization interface (light brown) and the C-terminus. Unfolded C-term: red. Ca^2+^-bound conformation: pink. Residues marked with asterisks represent the native-like conformation. Panel I shows the general movement of ∼2 Å in the catalytic domain, as well as the rearrangement of the loop (^156^NTFDGMYPY^164^). Dimer 1 with a native-like fold is shown in light blue and dimer 2 in dark blue. Panel II represents dimer 2 with an unfolded C-terminus (red) and loop-to-loop interaction in the dimerization interface (brown). Panel III shows the native-like conformation of dimer 1. The C-terminal conformation corresponding to the calcium-bound state is shown in pink with “modeled” Ca^2+^ binding (green). Helixes in the dimerization interface are colored light brown.

When comparing dimers 1 and 2 (PDB ID: 8PMR), major structural changes are visible not only in the secondary structure elements but also at the amino acid level. In dimer 2, the entire catalytic domain translocates ca. 2 Å away from the dimerization interface, also showing major rearrangements in the loop (^156^NTFDGMYPY^164^) containing residues Phe158 and Tyr164 (Figure 3, panel I)^8^ that were earlier observed to be important for substrate binding. Omit maps were calculated to confirm the correct placement of the loop in both dimer 1 and dimer 2 (Figure S4). At the same time, the unfolded C-terminus allows the α-helix of the opposite protomer at the dimerization interface to unwind into a loop conformation (Figure 3, panel II).

When the conformation of the C-terminus changes to “native-like” resembling the Ca^2+^-bound state in dimer 1 (Figure 3, panel III), the folding of the helix-turn-helix motif (harboring the Ca^2+^-binding site) forces the C-terminus to turn 45°. Simultaneously, residues Tyr224 and Lys221 adopt a new conformation toward the loop of the opposite protomer in the dimerization interface. This causes the loop to wind into a helical conformation. Thus, the formation of the Ca^2+^-binding site together with Tyr224 and Lys221 creates a “cap-like” structure to keep the helix folded like a compressed spring, harboring the Arg71_**B**_ (residues from opposite protomer marked with _**B**_) and Phe64 side chain of the corresponding protomer in it (Figure 3, panel III). The Lys221 side chain then forms a hydrogen bond with the Leu70_**B**_ main chain carbonyl. On the other side of the helix, Pro65 acts as a natural helix breaker.

It is reasonable to suggest that the formation of the, rather local, helix-turn-helix Ca^2+^-binding motif and the helical structure in the dimerization interface seem to induce a series of local structural changes that eventually lead to a tighter packing of the active site and create domain movement toward the dimerization interface. Key elements involved in this process include the movement of Tyr218 that causes a steric clash for Tyr162, which is forced to find a new conformation. This induces the loop ^156^NTFDGMYPY^164^ to change conformation away from the helix and makes space for Tyr164 (Figure 3, panel I). Local conformational changes are supported by circular dichroism data (Figure S3). Figure 3 illustrates the changes in the local conformation, and overall dynamics evoked by calcium binding are presented in the animation in the Supporting Information (Animation S1).

### *Af*NADase Ca^2+^ Binding Induces Conformational Changes that Optimize Active Site Geometry

**Figure 4:**
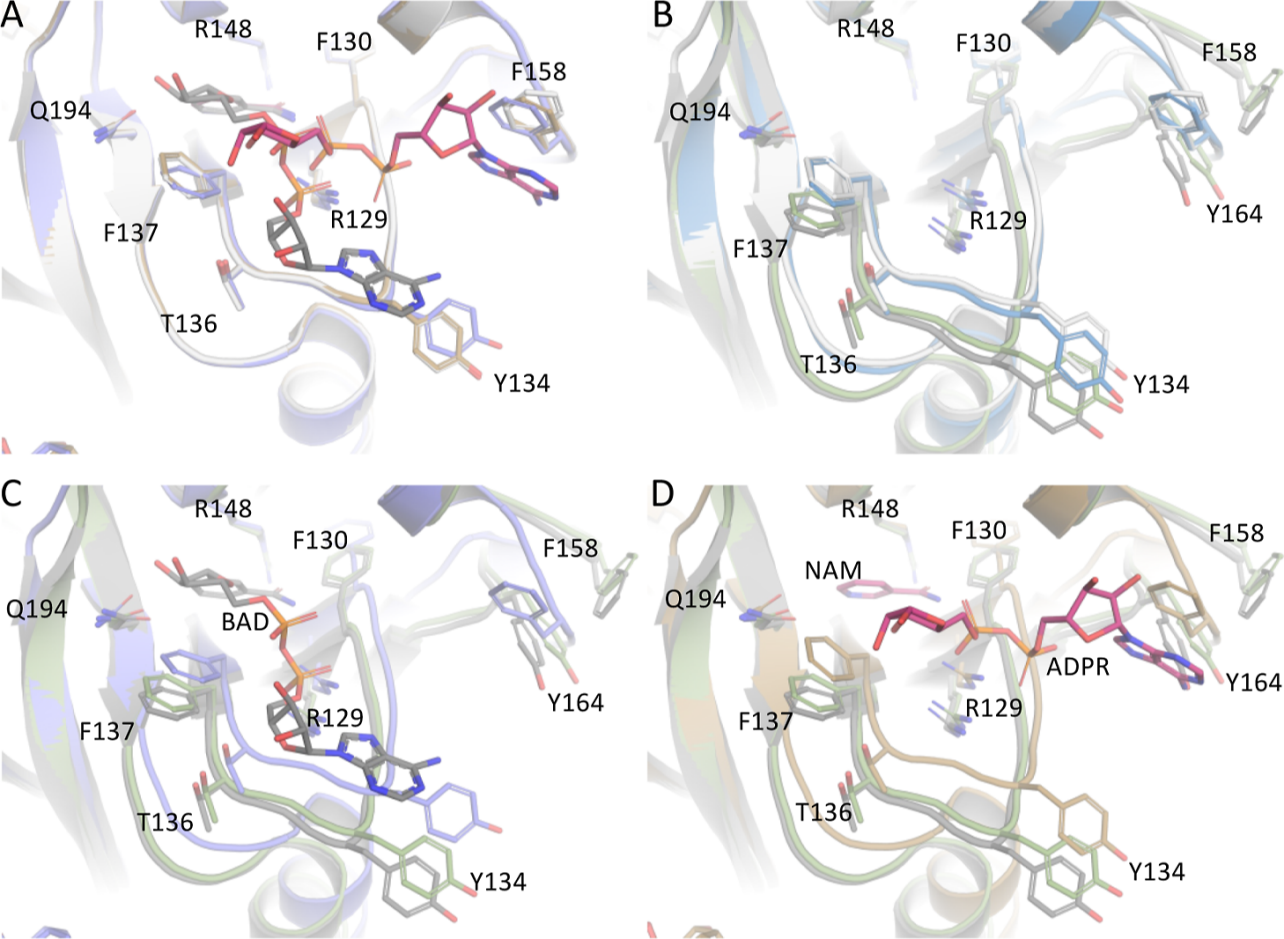
Comparison of possible Ca^2+^-regulated *Af*NADase active site conformations. (A) comparison of the published *Af*NADase structures of wt (light gray, PDB ID: 6YGE), wt with Nam and ADPR trapped in the active site (brown, PDB ID:6YGF), and wt with substrate analogue BAD bound to the active site (purple, PDB ID:6YGG) shows practically no differences in the active site geometry. (B) Comparison of wt (light gray), dimer 1 (blue, PDB ID:8PMR), dimer 2 (dark gray, PDB ID:8PMR), and *Af*NADase *Nc*^C-Term^ (green, PDB ID:8PMS) shows significant changes in the periphery of the active site. (C) Comparison of BAD-bound wt (purple), dimer 2 (dark gray), and *Af*NADase *Nc*^C-Term^ (green, ). Especially, changes in the Tyr134 and Phe137 conformations could have effects on guiding the substrate into the active site and orienting the Nam moiety correctly for efficient hydrolysis. (D) Comparison shows that in the state where the product ADPR is bound to wt (brown), the Phe158 interacts with the adenine moiety, and this interaction is not possible in dimer2 (dark gray) and *Af*NADase *Nc*^C-Term^ (green), where the loop undergoes rearrangements pushing Tyr164 to the position previously occupied by Phe158.

To investigate whether Ca^2+^ binding affects the active site geometry, the structure of the *Af*NADase^D219A/E220A^ dimers (PDB ID: 8PMR) was compared to previously published structures and to the *Af*NADase *Nc*^C-Term^ structure (PDB ID: 8PMS). The *Af*NADase *Nc*^C-Term^ crystal structure was solved and the description of the structure is presented in the Supporting Information (Table 2 and Figure S1B). The structure was chosen for the analysis based on the hypothesis that it represents a static conformation resembling dimer 2 of *Af*NADase^D219A/E220A^ (Figures S1C–S3). The active site of the wild-type *Af*NADase structures 6YGE, 6YGF, and 6YGG resembles the “native” active site conformation that also applies for dimer 1 of *Af*NADase^D219A/E220A^ (Figure 4A,B). It is noteworthy that the structure 6YGF, although being in native-like conformation, is lacking the Ca^2+^ from the Ca-binding motif due to experimental setup.^8^ When the active sites of dimer 2 and *Af*NADase *Nc*^C-Term^ are compared to the native conformation, we see that Arg148 in the bottom of the catalytic pocket, as well as Gln194, have retained their original position, while all other residues have shifted (∼2 Å). The most drastic change can be seen in loop ^156^NTFDGMYPY^164^, as already described above. The native conformation of the loop allows Phe158 to reach the orientation shown in the wild-type structures where it interacts with the product of enzyme catalysis (ADPR) in the active site (Figure 4A, PDB ID: 6YGF).^8^ Conversely, in dimer 2 and in the *Af*NADase *Nc*^C-Term^ structure, the loop ^156^NTFDGMYPY^164^ is observed in a new conformation described above (Figure 3, panel I). This conformation allows Phe158 to pull outside the catalytic pocket and therefore should be unable to interact with ADPR (Figure 4D). Moreover, the residues Thr136 and Phe137 are retracted from their native position, where they were detected to interact with the substrate analogue benzamide adenine dinucleotide (BAD, PDB ID:6YGG), indicating a more open conformation for the active site of dimer 2 (Figure 4C). Together, these observations show how Ca^2+^ binding affects the active site geometry. The more open conformation in the active site is likely to be the cause of the decreased catalytic activity of the mutant enzymes (Figure 1D).

## Discussion

This study has revealed a major regulatory component of *A. fumigatus* NADase, namely, the functional importance of binding of calcium to a recently identified C-terminal Ca^2+^-binding motif in the enzyme. Our analyses suggest that calcium binding evokes structural rearrangements in the dimer interface to stabilize the overall fold. In addition, local changes upon calcium binding are transmitted by long-range interactions to the catalytic site thereby greatly improving substrate affinity and turnover. As far as we are aware, this represents a novel mechanism of Ca^2+^-mediated enzyme regulation.

In many enzymes, metal ions are directly involved in catalysis by participating in electron transfer based on their redox potential or just bridging a substrate with the enzyme. Ca^2+^ specifically binds with a pentagonal bipyramidal coordination. Proteins containing the classical Ca^2+^ coordinating EF-hand, a 12 amino acid long loop flanked by 2 helixes, are known to participate in intracellular calcium homeostasis regulation and activation signaling pathways. For example, in troponin C, the Ca^2+^ binding results in conformational changes that trigger muscle contraction.^15^ Whereas, in thermolysin from *Bacillus thermoproteolyticus,* Ca^2+^ binding solely increases thermostability of the protein.^16^ In the case of *Af*NADase, Ca^2+^ is not located in the active site and therefore seems to be only a structural element. However, in this study, we show how *Af*NADase uses a unique Ca^2+^-binding motif to fine-tune protein folding, eventually increasing both structural stability and catalytic activity of the enzyme.

We recently observed that even EGTA-treated *Af*NADase shows only incomplete depletion of Ca^2+^ in the crystal structure. However, after soaking with NAD, the Ca^2+^ ion was entirely absent, while the reaction products (ADPR and Nam) were trapped in the active site (PDB ID:6YGF). As demonstrated here, calcium binding directly affects *Af*NADase activity (Figure 1C). In view of the observed Ca^2+^-dependent structural rearrangements, we posit that, besides its protein-stabilizing function (Figure 1F,G), the calcium binding motif has evolved to regulate the affinity toward the substrate NAD(P)^+^ or increase the rate of product release, resulting in an accelerated catalytic rate (Figure 1D).

Crystal structures represent a rigid snapshot of a protein structure in solution; therefore, it is normally necessary to determine multiple crystal structures under different conditions to gain insights into the dynamic properties of a protein. Alternatively, complementary methods, such as SAXS and NMR can be used. Wild-type *Af*NADase crystallizes as a biological dimer in the ASU, but remarkably, *Af*NADase^D219A/E220A^ (PDB ID:8PMR) has two dimers with different conformations present in the ASU. Thus, dynamic counterparts can be identified from a single crystal structure. These can be thought to represent the native, Ca^2+^-bound state of *Af*NADase (dimer 1) and the situation where the Ca^2+^ ion would have been depleted and the C-terminus is therefore unfolded (dimer 2). Accordingly, the *Af*NADase *Nc*^C-Term^ structure (PDB ID: 8PMS), which lacks the Ca^2+^-binding motif, resembles the dimer 2 structure.

Based on the biochemical and structural data, it would appear that wild-type *Af*NADase predominantly, if not exclusively, exists in the conformation stabilized by Ca^2+^ binding. Conversely, *Af*NADase^D219A/E220A^ seems to have a dynamic equilibrium in solution between dimer 1 and dimer 2 (Figure S3), resulting in both lowered thermal stability and decreased catalytic activity when compared to wild-type *Af*NADase. In line with this suggestion, the *Af*NADase *Nc*^C-Term^ variant (dimer 2-like), which lacks the ability to form the native-like C-terminal conformation, exhibits substantially decreased catalytic activity and thermostability. That is, it is reasonable to assume that *Af*NADase *Nc*^C-Term^ (PDB ID:8PMS) represents a static “dimer 2-like” conformation (Figures S1B,C and S3). These considerations support the role of calcium as a conformational stabilizer to attain an optimized active site geometry.

In addition to increased catalytic activity, the binding of calcium increases thermal stability (Figure 1E–G). Most Eurotiales species, including *A. fumigatus*, are reported to be thermophilic fungi.^17^ The presence of this unique Ca^2+^-binding motif, found solely in this order of fungi,^8^ might suggest that it is an evolutionary trait that enhances thermostability and catalytic activity when compared to other fungal orders.

We have shown that when calcium is unable to bind the protein adopts an alternative fold with a more open active site. The higher *K*_M_ can be explained by this enlarged active site conformation when comparing the wild-type *Af*NADase complexed with BAD to the dimer 2 structure of the *Af*NADase^D219A/E220A^ variant (Figure 4C). Tyr134 and Phe137, which are responsible for the aromatic interactions coordinating the substrate, are retracted further away from Arg148 and Gln194 in dimer 2 of *Af*NADase^D219A/E220A^ that remain static. Arg148 interacts with the Nam moiety at the bottom of the active site, whereas Gln194 is suggested to be a key mediator of NAD^+^ hydrolysis by initiating the reaction with a nucleophilic attack on the 2” OH- on the proximal ribose of NAD^+^.^8^ In addition, Arg129 reorients in concert with the other substrate-interacting residues, probably making the substrate binding weaker. One could hypothesize that, upon NAD^+^ hydrolysis, wild-type *Af*NADase could speed up the product release by altering its conformation, dependent on the occupancy of the Ca^2+^-binding site. In this case, Phe158 and Tyr164 could interact with the adenosine moiety pulling the ADPR out from the pocket assuming a conformation similar to dimer 2 and *Af*NADase *Nc*^C-Term^. Indeed, as previously shown, Phe158 interacts with adenosine in the *Af*NADase structure complexed with reaction products (PDB ID: 6YGF). However, factors regulating the occupancy of the Ca^2+^-binding site have yet to be identified.

## Summary

The study suggests a functional role of a Ca^2+^-binding motif that represents a characteristic trait of Eurotiales NADases. Calcium binding causes the dimerization interface of the enzyme to form a more compact conformation that positively affects protein stability and glycohydrolase activity. To our knowledge, this motif represents a hitherto undescribed strategy to promote and stabilize protomer interactions. Moreover, we show that the motif affects the catalytic activity of the enzyme through a major reconfiguration of the dimerization interface which is ultimately transmitted to rearrange the catalytic pocket conformation. The absence of this binding motif in other divisions of the fungal kingdom (e.g., *Sordariomycetes* including *N. crassa*) suggests that it represents an evolutionary trait that may be important for thermophilic species such as *A. fumigatus*. Mechanistic insights into the catalytic functions and regulation of TNT-like fungal NADases may eventually help to develop new concepts to combat fungal infections.

## Materials and Methods

### Expression and Purification of *Af*NADase, *Af*NADase^D219A/E220A^, and *Af*NADase *Nc*^C-Term^

The plasmids and the viruses encoding the *Af*NADase, *Af*NADase^D219A/E220A^ variant, and *Af*NADase *Nc*^C-Term^ were generated as described in Strømland et al.^8^ and following the protocol of Bieniossek et al. for bacmid and baculovirus propagation.^18^ For protein expression, Sf9 cells were cultivated in Sf-900 II SFM (Gibco) medium to a density of 1.2–1.5 million cells/mL before infection with a high titer of viral stocks. Cells were cultivated for another 3 days after infection and then pelleted by centrifugation. The supernatant containing the secreted enzyme was filtered using a 0.22 μM filter (Merck Millipore) and purified by immobilized metal affinity chromatography using HisTrap excel (GE Healthcare) column connected to an ÄKTA pure chromatography system (GE Healthcare). Washing was performed with washing buffer (50 mM Tris-HCl pH 8.0, 300 mM NaCl) and elution with elution buffer (50 mM Tris-HCl pH 8.0, 300 mM NaCl, 500 mM Imidazole). The collected fraction was concentrated with 10 kDa MWCO Amicon Ultra centrifugal filters (Merc Millipore) and imidazole was removed using a PD-10 desalting column (Cytivia). The sample was then incubated overnight with 3Case to cleave the 6xHis-tag, and the 3Case was removed with a batch of His-affinity chromatography. Size exclusion chromatography was performed as the final purification step using a Superdex 200 16/60 HiLoad prep grade column (GE Healthcare), using 50 mM Tris-HCl pH 8.0, and 300 mM NaCl as the final elution buffer. For all purified enzymes, the yield was around 8–10 mg per liter of medium.

### Generation of *Af*NADase *Nc*^C-Term^

The chimeric protein was generated using a PCR-based strategy by mutation of the parental plasmids generated by Strømland et al.^8^ using a Q5 Site-Directed Mutagenesis Kit (New England Biolabs Inc.). The primers were designed using the NEB base exchanger web tool (New England Biolabs Inc.). *Af*NADase *Nc*^C-Term^ was designed using the primers 5′-gccacggaactacggtaccGACGTGCTGTTTCAGGGC and 5′- accagtctctgtgggtcttcCCGTCGCAAGTAACCATC following Gibson assembly protocol.^19^

### Determination of *Af*NADase, *Af*NADase^D219A/E220A^, and *Af*NADase *Nc*^C-Term^ Kinetics by Fluorescence Spectrophotometry

The kinetic characterization of the enzymes in the study was conducted using a TECAN Spark multimode plate reader, following the hydrolysis of a fluorescent NAD analogue (etheno-NAD, excitation–emission wavelength 300–410 nm, respectively) in 96-well black flat-bottom plates (Corning) at 25 °C. The kinetic measurement was conducted with 10 ng of enzyme and a substrate concentration ranging from 1 to 500 μM in reaction buffer (50 mM sodium acetate pH 5.5, 150 mM NaCl, 500 μM CaCl_2_) in a final volume of 100 μL. Natural occurring degradation of etheno-NAD was monitored and subtracted from the values obtained from the enzymatic reactions. Relative fluorescence units (RFUs) were converted into moles using a calibration curve obtained from the complete conversion of different concentrations of etheno-NAD upon 2 h incubation with 100 ng of purified *Af*NADase. The results were background-corrected against the substrate alone in the reaction buffer. The kinetic parameters were calculated using GraphPad Prism 9.1.1 and plotted using the Michaelis–Menten equation.

### Circular Dichroism

Circular dichroism (CD) was performed using a Jasco J-810 spectropolarimeter with a protein concentration of 0.1 mg/mL for all three purified enzymes (*Af*NADase, *Af*NADase^D219A/E220A^, and *Af*NADase *Nc*^C-Term^). Spectra were acquired between 280 and 180 nm with a wavelength step of 0.5 nm, scan speed of 50 nm/min, bandwidth of 1 nm, accumulation 3, and N_2_-flow of 10 L/min. The results were evaluated using DichroWeb,^20^ CONTIN analysis program, and SMP180t reference set.

### Crystallization and Structure Determination

Prior to crystallization screening, *Af*NADase^D219A/E220A^ was concentrated using an Amicon Ultra centrifugal filter (Merck KGaA, Darmstadt, Germany) with a 10 kDa cutoff. Crystallization experiments were done using the vapor diffusion method with MRC SD2 sitting drop plates (Molecular Dimensions Limited, Rotherham, UK), and the liquid handling was performed with a Mosquito LCP crystallization robot (SPT LabTech, Melbourn, UK).

The initial screenings were carried out using the following commercial screens: PACT premier, JSCG plus, and MemGold at +20 and +8 °C. Hits were optimized at +20 °C, resulting in crystals after 3–5 days from a condition containing 0.2 M NaCl, 0.1 M MES pH 6.0, and 20% PEG 6000.

The screening for crystallization conditions was done in a similar manner for the *Af*NADase *Nc*^C-Term^. Hits were again optimized at +20 °C, resulting in crystals from conditions containing 0.02 M MgCl_2_, 0.1 M HEPES, and 22% polyacrylic acid 5100.

Crystals were cryoprotected by soaking in a crystallization solution supplemented with 30% glycerol prior to flash freezing in liquid N_2_. Diffraction data for *Af*NADase^D219A/E220A^ were collected at 100 K by using synchrotron radiation (λ = 0.976 Å) at the P13 beamline at PETRA III/EMBL in Hamburg, Germany. The data for *Af*NADase *Nc*^C-Term^ were collected at 100 K by using synchrotron radiation (λ = 1.033 Å) at the P11 beamline at PETRA III/DESY in Hamburg, Germany. Diffraction data were processed using X-ray Detector Software (XDS)^21^ and scaled using AIMLESS.^22^ Crystals belonged to space groups *P*6_5_ and *P*4_1_2_1_2 for *Af*NADase^D219A/E220A^ and *Af*NADase *Nc*^C-Term^, respectively (Table 2). The structures were determined using the molecular replacement in phaser^23^ and the *Af*NADase (PDB-ID: 6YGE)^8^ as a search model. The structures were refined with phenix.refine^24^ in PHENIX-package^25^ in alternating cycles with manual adjustment performed in Coot.^26^ Illustrations for the figures were created in PyMol (The PyMOL Molecular Graphics System, Version 2.0 Schrödinger, LLC.). The diffraction and refinement statistics are shown in detail in Table 2.

### EGTA/Ca^2+^ Sensitivity Assay and Residual NADase Activity

The metal ion sensitivity assay was performed as reported for the kinetic characterization of the NADases but with the addition of increasing concentration of EGTA or Ca^2+^ in the reaction buffer (50 mM sodium acetate, pH 5.5, 150 mM NaCl) and a fixed substrate concentration of 80 μM. The results were background-corrected against the substrate alone in the reaction buffer and plotted using GraphPad Prism.

Residual NADase activity evaluation was performed after treating the incubation buffer (50 mM Tris-HCl pH 8.0, 300 mM NaCl) with Chelex 100 chelating ion exchange resin, in order to minimize the presence of unadded Ca^2+^ and other ions in the buffer. Incubation was conducted either with 2 mM EGTA or 2 mM Ca^2+^ at RT and 50 °C for 10 min. Residual activity was evaluated as reported for kinetic characterization of the NADases by adding 50 ng of the enzyme from each incubation reaction in reaction buffer (50 mM sodium acetate, pH 5.5, 150 mM NaCl).

### DSF to Determine *T*_M_

Thermal denaturing was investigated to obtain the protein melting point, *T*_M_. Denaturing profiles for the enzymes were recorded using a Light Cycler 480 Real-Time PCR (Roche) with a 0.2 mg/mL protein concentration in phosphate-buffered saline supplemented with 5x SYPRO Orange (Thermo) on 384-well plates with 20 μL sample volume. The effect of Ca^2+^ and EGTA on the *T*_M_ of the NADases was tested at a concentration of 5 mM for both molecules. Incubation of *Af*NADase and *Af*NADase *Nc*^C-Term^ with Ca^2+^ and EGTA was performed at a concentration of 5 mM. The results were background-corrected against the substrate alone. The data were analyzed using HTSDSF Explorer^27^ and GraphPad Prism.

## Supporting Information Available

Structural differences of *Af*NADase *Nc*^C-Term^; crystal packing and crystal contacts analysis of *Af*NADase^D219A/E220A^; CD spectra of *Af*NADase, *Af*NADase^D219A/E220A^, and *Af*NADase Nc^C-Term^; and omit maps calculated for dynamic loop ^156^NTFDGMYPY^164^ (PDF)
Animation of Ca ^2+^-mediated *Af*NADase dynamic conformational changes (MP4)

## Accession Codes

Deposition to RCSB PDB for coordinates and structure factors of *Af*NADase^D219A/E220A^ and *Af*NADase *Nc*^C-Term^ were done under accession codes 8PMR and 8PMS, respectively.
