# Supplementary material for: Novel Calcium-Binding Motif Stabilizes and Increases the Activity of Aspergillus fumigatus Ecto-NADase
Source: Biochemistry. 2023 Nov 7;62(22):3293–302. doi: 10.1021/acs.biochem.3c00360 (PMC10666276; doi:10.1021/acs.biochem.3c00360)
Supplement: Supplementary file 1 — bi3c00360_si_001.pdf [file bi3c00360_si_001.pdf]

Supporting Information

A novel calcium binding motif stabilizes and increases the activity of *Aspergillus fumigatus* ecto-NADase

Eugenio Ferrario[1†], Juha P. Kallio[1†], Øyvind Strømland[1], Mathias Ziegler[1,2*]

[1] Department of Biomedicine, University of Bergen, Jonas Lies vei 91, 5009 Bergen, Norway

[2] Leibniz Institute for Natural Product Research and Infection Biology, Hans Knöll Institute, Beutenbargstraße 11A, 07745, Jena, Germany

* Corresponding author: Mathias Ziegler, Mathias.Ziegler@uib.no

† Both authors contributed equally to this work

Supplementary information includes:

Figure S1. Structural differences of *Af*NADase *Nc*$^{\text{C-Term}}$

Figure S2. Crystal contacts analysis of *Af*NADase$^{\text{D219A/E220A}}$

Figure S3. Circular dichroism spectra of *Af*NADase, *Af*NADase$^{\text{D219A/E220A}}$, and *Af*NADase *Nc*$^{\text{C-Term}}$

Figure S4. Omit maps calculated for dynamic loop ^{156}NTFDGMYPY^{164}

Animation S1. Animation of Ca^{2+} mediated *Af*NADase dynamic conformational changes

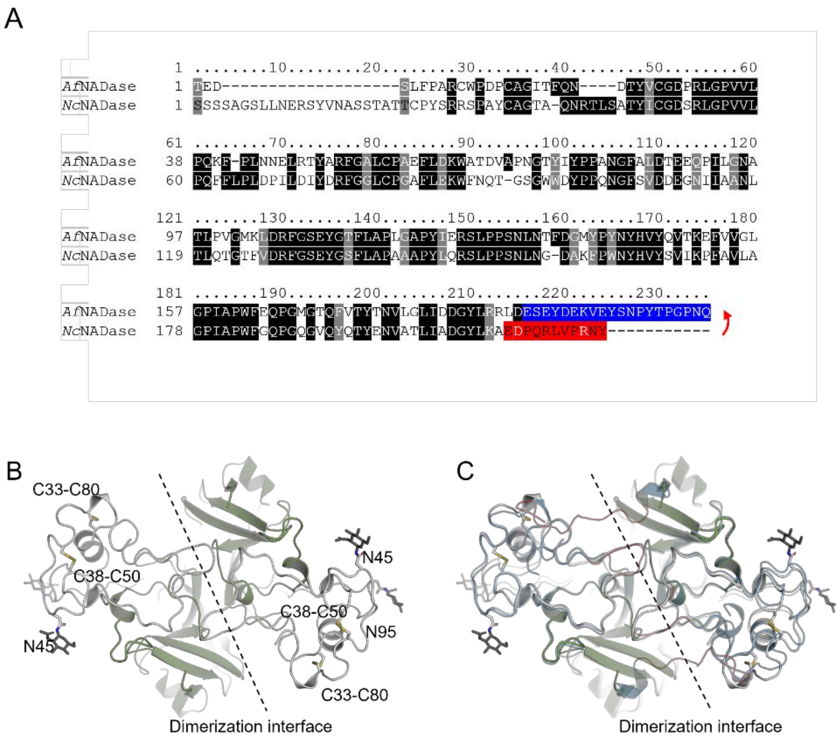

Figure S1. Structural differences of *Af*NADase *Nc*$^{\text{C-Term}}$. A) Sequence alignment of *Af*NADase and *Nc*NADase with highlighted in blue the C-term portion of *Nc*NADase used to replace the Ca^{2+} binding motif (red) in *Af*NADase *Nc*$^{\text{C-Term}}$. B) Cartoon representation of the biological dimer of *Af*NADase *Nc*$^{\text{C-Term}}$ (PDB ID: 8PMS). Catalytic domain is presented in green, and the palm domain in grey. C-terminus was not detectable in the electron density and therefore not built into the model C) Comparison of dimer 2 of Ca^{2+}-variant (PDB ID: 8PMR) to *Af*NADase *Nc*$^{\text{C-Term}}$ (8PMS) reveals that *Af*NADase *Nc*$^{\text{C-Term}}$ structure represents the conformation with unfolded C-terminus. Only small differences in the palm domain can be detected.

Structure description of *Af*NADase *Nc*$^{\text{C-Term}}$

With this construct we aimed to understand how the complete substitution of the C-terminus containing the Ca^{2+} binding motif might affect *Af*NADase. *Af*NADase *Nc*$^{\text{C-Term}}$ purified as a stable dimer, like the other constructs. The *Af*NADase *Nc*$^{\text{C-Term}}$ was crystallized and the structure determined in space group $P4_12_12$ with a resolution of 2.4Å and showing 4 molecules in the ASU (two dimers) (PDB ID: 8PMS). The overall structure of both dimers was similar to what we saw with dimer 2 of the *Af*NADase$^{\text{D219A/E220A}}$. Albeit, the electron density at both C-term of the protomers was not visible, assumingly because of the intrinsic flexibility of the region. Double conformation in the loop located in the dimerization interface was detected in one of the dimers but this did not have any effect on the overall fold of the protein. The dimerization interface resembles the dimer 2 (PDB ID: 8PMR) state of *Af*NADase$^{\text{D219A/E220A}}$.

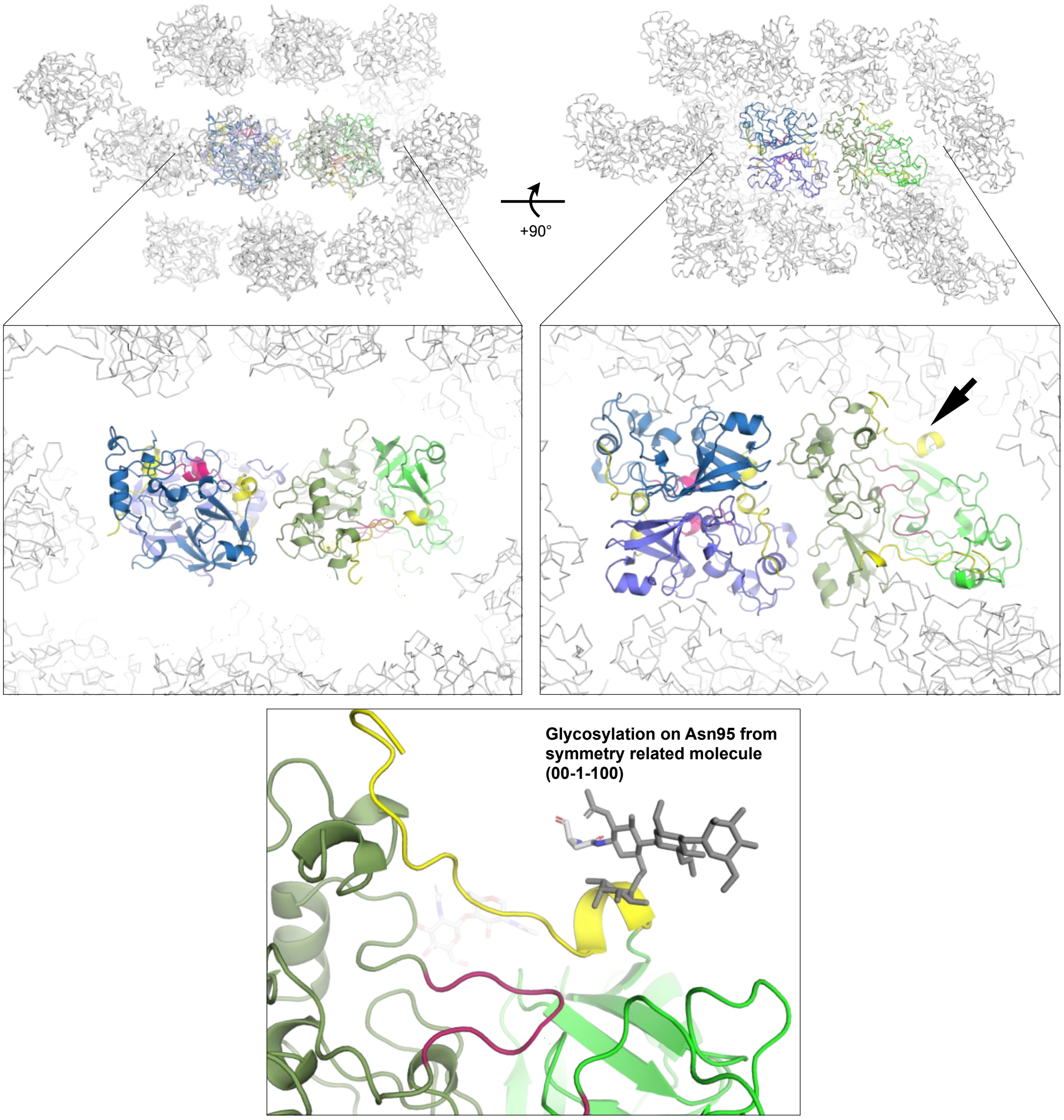

FIGURE S2. Crystal packing and crystal contacts analysis of *Af*NADase$^{D219A/E220A}$. The 2 dimers in the ASU (asymmetric unit) are shown in blue (dimer 1) and green (dimer 2), and the symmetry related dimers in grey (PDB ID: 8PMR). Secondary structure elements at the dimerization interface are shown in pink and the C-terminal tail in yellow. *Af*NADase$^{D219A/E220A}$ crystallizes in the space group P6$_5$. Crystal packing around the dimerization interfaces is rather loose, leaving a lot of solvent-accessible space between the ASU and symmetry-related molecules. This space is highlighted in orange. There is no indication of crystal contacts that would force the conformation to either direction. When the view is rotated 90° on the x-axis, more tight packing can be detected, however, no crystal contacts that would force the conformation to either direction were detected. The closest contact facing towards the C-terminal tail of dimer 2 (indicated with black arrows) is shown in the last panel. This shows N-glycosylation from Ans95 from a symmetry related molecule to pack close to residues ^{214}DESEY^{218}. No clashes were found between ASU and symmetry related molecules.

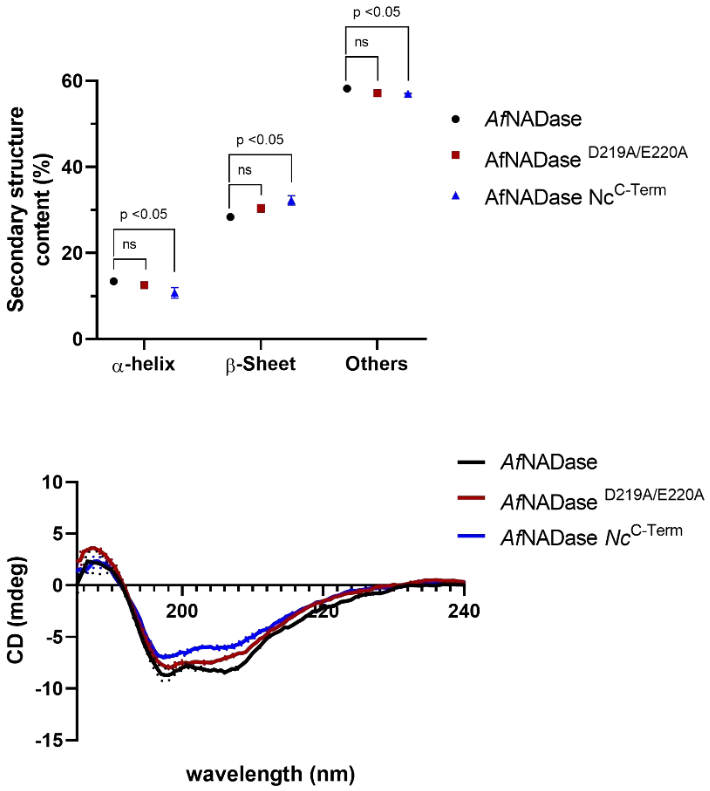

Figure S3. Circular dichroism spectra of *Af*NADase, *Af*NADase$^{\text{D219A/E220A}}$, and *Af*NADase *Nc*$^{\text{C-Term}}$. Circular dichroism (CD) spectra of the three NADases show different helical content (*Af*NADase 13.43% ±0.15 n=3; *Af*NADase$^{\text{D219A/E220A}}$ 12.53% ±0.57 n=3; *Af*NADase *Nc*$^{\text{C-Term}}$ 10.75% ±1.2 n=3), following what described by the crystal structures (6YGE, 8PMR, 8PMS). The experiment was performed with Jasco J-810 spectropolarimeter, using a protein concentration of 0.1 mg/mL and acquiring 280-180nm (wavelength step 0.5nm). Results have been evaluated using DichroWeb, using CONTIN analysis program, and SMP180t reference set. Assays were performed in technical triplicates. Error bars represent standard deviation.

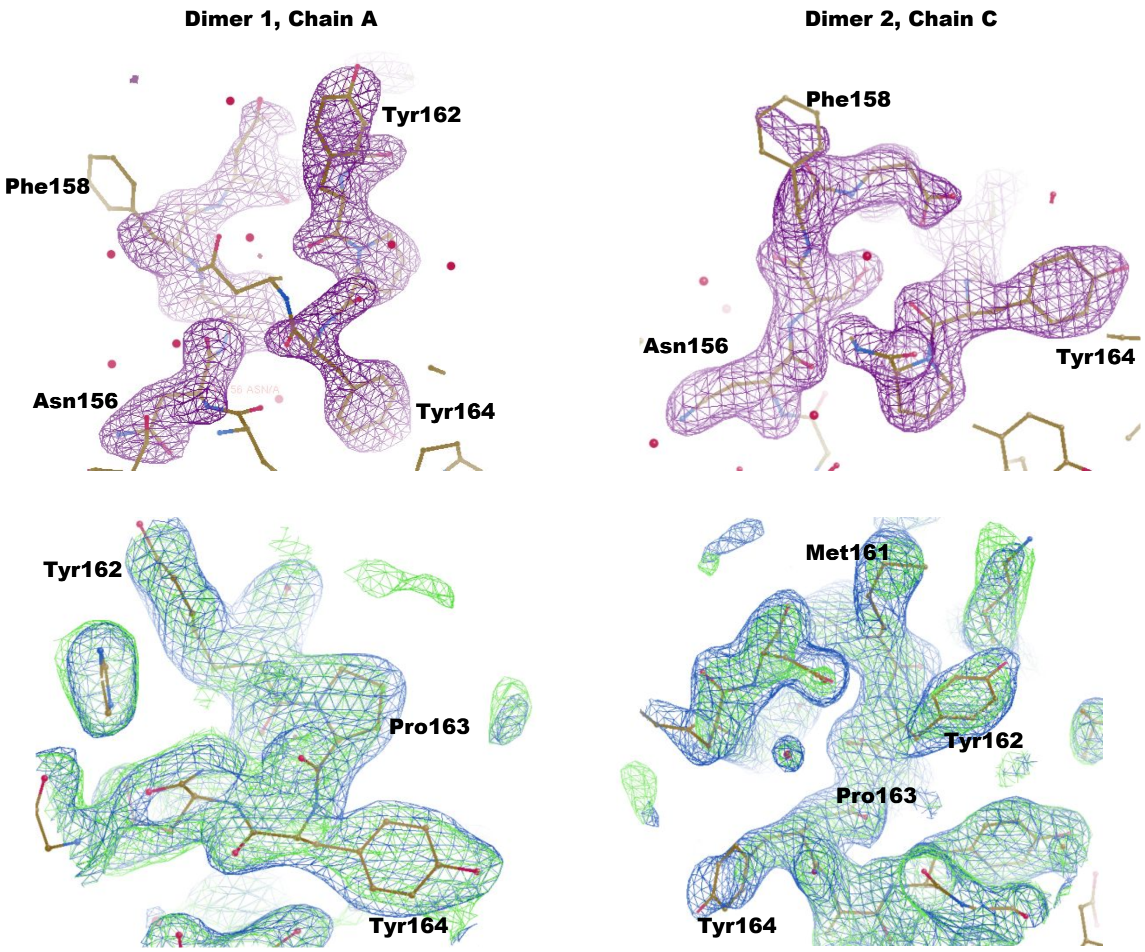

Figure S4. Omit maps calculated for dynamic loop ^{156}NTFDGMYPY^{164}. Maps were calculated by omitting loop residues and using phenix.composite_omit with cartesian annealing. Purple mesh represents the mFo-DFc map at contour level 5σ. Blue mesh is the 2mFo-DFc map from refinement and green mesh is the corresponding omit map. Both are represented with contour level 1σ. Omit mFo-DFc map gives a justification for the correct placement of the loop residues during refinement. High degree of map similarity in the 2mFo-DFc maps indicates that refined map does not carry significant model bias.

Animation S1. Animation of Ca^{2+} mediated *Af*NADase dynamic conformational changes. Morph conformation animation describing the structural transition from unbound (PDB ID: 8PMR) to Ca^{2+} bound state (PDB: 6YGE) of *Af*NADase. The animation tracks the trajectory that morphs between the two crystal structures 8PMR and 6YGE, dynamically highlighting the conformational changes at the dimerization interface, Ca^{2+} binding motif, and catalytic pocket of *Af*NADase.
